# Individual Differences in Growth and in Accumulation of Secondary Metabolites in *Rhodiola rosea* Cultivated in Western Siberia

**DOI:** 10.3390/ijms241411244

**Published:** 2023-07-08

**Authors:** Anna A. Erst, Olga V. Kotsupiy, Andrey S. Erst, Alexander A. Kuznetsov

**Affiliations:** 1 Central Siberian Botanical Garden, Siberian Branch of Russian Academy of Sciences, Novosibirsk 630090, Russia; olnevaster@gmail.com (O.V.K.); erst_andrew@yahoo.com (A.S.E.); 2 Laboratory Herbarium (TK), Tomsk State University, Tomsk 634050, Russia; ys.tsu@mail.ru

**Keywords:** HPLC, roseroot, individual variability, rosavins, salidroside, tyrosol, flavonoids, hydroxybenzoic acids, catechins, Altai Mountains

## Abstract

In this study, growth parameters of underground parts and concentrations of phenylpropanoids, phenylethanoids, flavonoids, hydroxybenzoic acids, and catechins in aqueous–ethanol extracts of 6-year-old cultivated plants of *Rhodiola rosea* (propagated in vitro) of Altai Mountain origin were analyzed, and differences in chemical composition among plant specimens and between plant parts (rhizome and root) were evaluated. High-performance liquid chromatography detected 13 phenolic compounds. Roots contained 1.28 times higher phenylethanoids levels (1273.72 mg/100 g) than rhizomes did. Overall, the concentration of phenylethanoids in underground organs was not high and ranged from 21.36 to 103.00 mg/100 g. High variation among *R. rosea* individual plants was noted both in growth characteristics and in levels of secondary metabolites under our cultivation conditions. It was found that concentrations of phenylpropanoids, phenylethanoids, and catechins significantly depend on the plant part analyzed (*p* ≤ 0.05). Specimen No. 4 is characterized by the highest concentration of rosavins (1230.99 mg/plant) and the lowest concentration of cinnamyl alcohol (62.87 mg/plant). Despite the wide range of values, all 10 tested specimens (underground part) met the minimum requirements of the United States Pharmacopeia (2015) for rosavins (0.3%) and of the Russia State Pharmacopoeia (2015) for the average level of rosavins (roots): (1%).

## 1. Introduction

According to the literature, approximately 140 organic compounds have been isolated in *Rhodiola rosea* L. (Crassulaceae): polyphenols, organic acids, sugars, tannins, terpenes, and essential oils [1,2,3,4]. The plant contains also so-called marker compounds characteristic of this species: phenylpropanoids [rosavins (rosavin, rosin, and rosarian) and cinnamyl alcohol] and phenylethanoids [salidroside, viridoside, and tyrosol]. Peter Zomborszky et al. [5] have hypothesized that flavonoids (rhodiosin and herbacetin) may serve as an additional marker to ensure consistent composition of *R. rosea* products. The United States Pharmacopeia allows 0.3% rosavins and 0.08% salidroside [6]; according to the Russia State Pharmacopoeia, the rosavin content must be not less than 1% and not less than 0.8% in terms of salidroside [7]; the Australian standard for the extract is not less than 1.8% phenylpropanoids, 1.2% rosavin, and 0.6% salidroside (https://www.tga.gov.au/resources/resource/compositional-guidelines/dried-root-powdered-rhodiola-rosea; accessed on 2 June 2023). Pharmacological studies on *R. rosea* are numerous [1,8,9,10,11,12,13,14,15]. Adaptogenic properties of *R. rosea* are mostly related to the presence of these marker compounds [16], while antioxidant activity is mainly due to organic acids and flavonoids [17].

Earlier studies have addressed effects of which plant part is analyzed, plant age, plant origin, plant sex, harvesting time, and cultivation methods on roseroot phytopharmaceuticals [18,19,20,21,22,23,24,25,26]. For instance, for cultivated *R. rosea* of European origin, it has been shown that the level of rosavins and salidroside depends more on the harvest season, age, and the assayed plant part than on the sampling site [21,22]. Peschel et al. [23] recommend harvesting *R. rosea* in the spring before or during emergence from soil, when the level of rosavins is at its highest. Substantial seasonal fluctuations of the levels of rosavins and salidroside were noted when the plants were cultivated under controlled artificial conditions of a phytotron, with the highest yield of these substances seen at the beginning of the growing season [27]. Rybakova et al. [28] have investigated the effect of spectral composition of light on *R. rosea* cultivation: in terms of both biomass productivity and the yield of salidroside per unit area, red light turned out to be the most effective. Some authors have demonstrated that the extraction method is of paramount importance for quantitative parameters of roseroot’s secondary metabolites, including rosavins and salidroside [29,30,31].

Global commercial demand for *R. rosea* is almost exclusively satisfied by wild harvested plants. *R. rosea* is very popular among local peoples, resulting in uncontrolled harvesting of this plant and, hence, to depletion of natural wild populations. Currently, *R. rosea* is listed as an endangered species and included in the Red Book of the Russian Federation [32]. The species is recommended for inclusion into the IUCN Red List of Threatened Species. The global demand for *R. rosea* raw material will continue to grow [33], which could lead to catastrophic consequences [34]. Natural reserves of *R. rosea* are being depleted, and therefore the development of in vitro propagation and conservation methods for most promising specimens from the Altai Mountains is an extremely urgent task, as is assessment of biosynthetic potential and biological productivity upon introduction.

The purpose of the study was to investigate interindividual variation in growth parameters and of concentrations of secondary metabolites as well as the productivity of *R. rosea* plants (originating from the Altai Mountains) cultivated using in vitro clones under the conditions of the forest–steppe zone of Western Siberia.

## 2. Results

### 2.1. Growth Parameters

**Figure 1:**
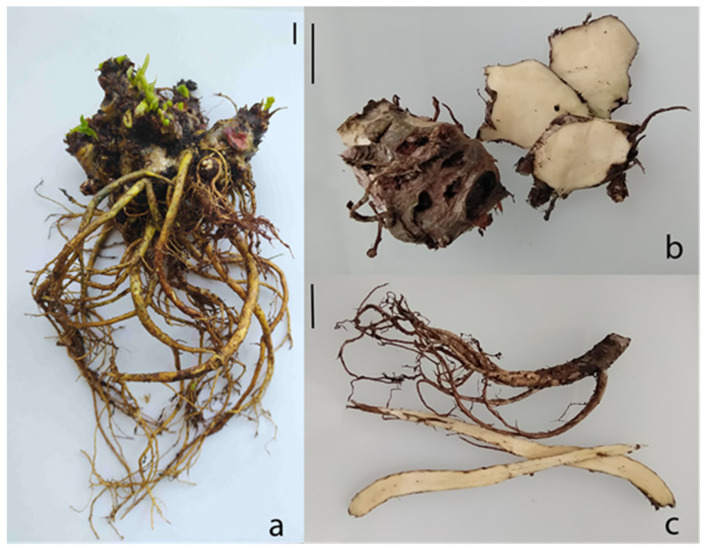
A 6-year-old *R. rosea* plant (**a**) grown in the forest–steppe zone of Western Siberia (Russia): parts of the rhizome (**b**) and root (**c**) used for biochemical analysis. Scale bars: 1 cm.

The largest dry biomass (dry weight) of roots and rhizomes was registered in the 6th year of cultivation and was 71.56 ± 12.72 g (Figure 1, Table 1). The rhizome:root ratio (dry weight) and biomass growth varied over the years. The highest rhizome:root ratios and the largest increase in biomass were observed in the 4th and 6th years of cultivation (1:0.58 and 362% and 1:0.44 and 315%, respectively). Overall, the mass of the rhizome always exceeded that of the roots, and the rhizome:root ratio varied from 1:0.44 to 1:0.78. Belowground biomass (total dry weight) variability was high (range 52.92–126.81 g, coefficient of variance 17.78%).

### 2.2. Analysis of Phenolic Compounds

**Figure 2:**
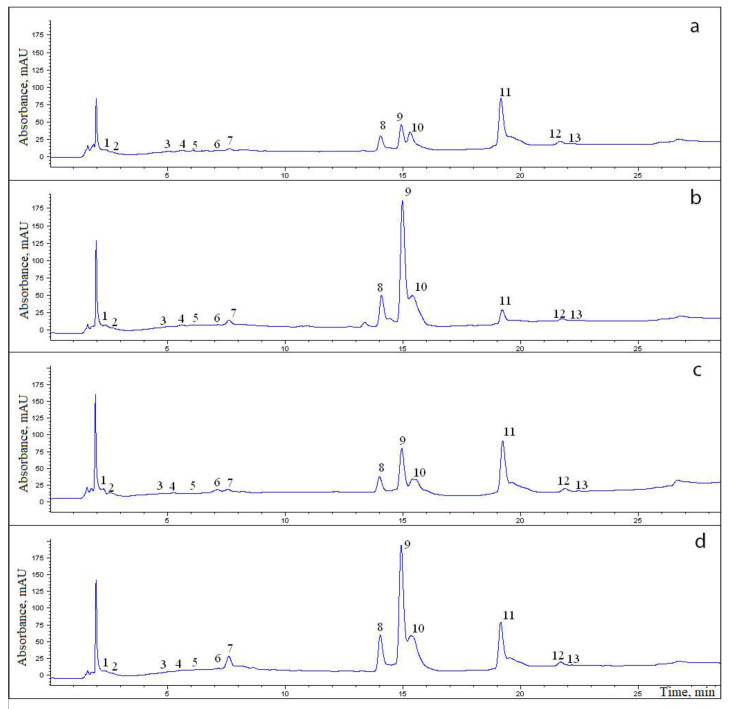
Chromatograms of an aqueous–ethanol extract of the rhizomes (**a**,**b**) and roots (**c**,**d**) of specimens No. 1 (**a**,**c**) and No. 4 (**b**,**d**) of *R. rosea*. The numbers in the chromatogram denote ID numbers of compounds corresponding to the ID numbers of compounds in Table 2.

Via high-performance liquid chromatography (HPLC), 13 phenolic compounds were found in the roots and rhizomes of *R. rosea* (Table 2, Figure 2). The average concentration of phenolic compounds did not differ significantly between the rhizome and root and was 1429.27 ± 113.52 mg/100 g (coefficient of variance 25.13%) and 1762.61 ± 173.59 mg/100 g (coefficient of variance 31.13%), respectively.

### 2.3. The Phenylpropanoid Content

**Figure 3:**
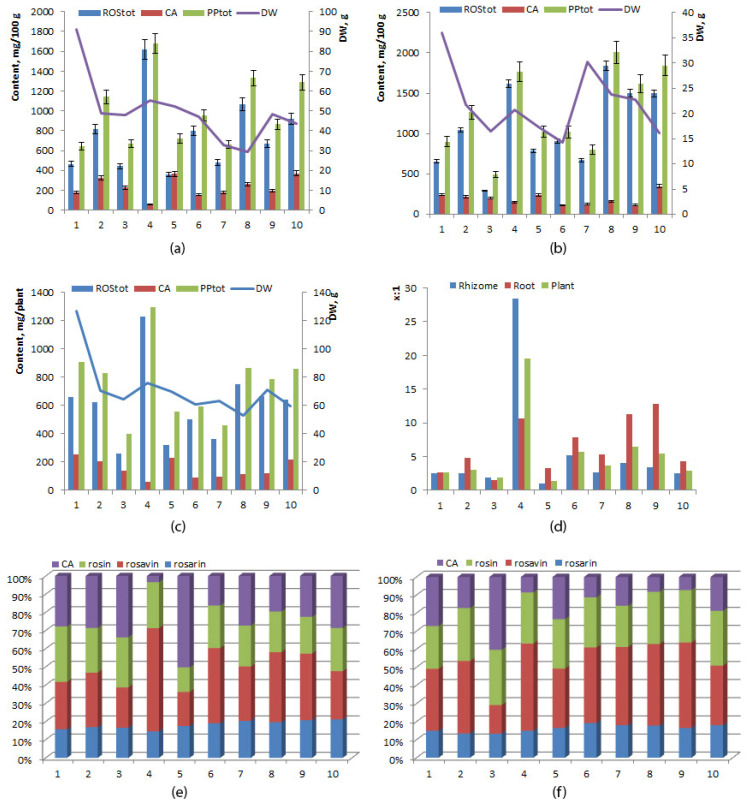
Concentrations of rosavins, cinnamyl alcohol, and phenylpropanoids in rhizomes (**a**), in roots (**b**), in the whole plant; (**c**) rosavins/cinnamyl alcohol ratio (x:1) (**d**) and a relative phenylpropanoid profile (%) of the rhizome (**e**) and root (**f**) of 6-year-old *R. rosea* plants cultivated in the forest–steppe zone of Western Siberia. The horizontal axis: plants (specimens) No. 1–10.

The concentration of phenylpropanoids and rosavins was on average significantly higher in the root than in the rhizome (1273.72 ± 160.65 and 1081.43 ± 159.89 mg/100 g versus 994.76 ± 111.59 and 763.60 ± 120.25 mg/100 g, respectively) (Table 2). The mean level of cinnamyl alcohol did not differ significantly between the rhizome and root (231.16 ± 31.50 and 192.29 ± 23.01 mg/100 g, respectively). Simultaneous analysis of samples from 10 individual plants showed that interindividual variation in levels of rosavins and cinnamyl alcohol was high in both the root and rhizome: in the root, rosavins ranged between 293.65 and 1841.91 mg/100 g (coefficient of variance 46.75%), and cinnamyl alcohol ranged between 115.21 and 346.37 mg/100 g (coefficient of variance 37.85%), whereas in the rhizome, rosavins ranged between 358.01 and 1650.10 mg/100 g (coefficient of variance 49.80%), and cinnamyl alcohol ranged between 56.96 and 369.01 mg/100 g (coefficient of variance 43.10%) (Figure 3a,b). No direct or inverse correlation was found between the yield of roots, of rhizomes, or of the whole plant and the concentration of phenylpropanoids (*p* ≤ 0.05). The highest level of rosavins was detected in specimen No. 4 (1230.99 mg/plant, dry weight 76.04 g), and the lowest in specimen No. 3 (259.56 mg/plant, dry weight 64.26 g). Specimen No. 1 manifested the highest cinnamyl alcohol content (251.07 mg/plant, dry weight 126.81 g), whereas cinnamyl alcohol content was the lowest in specimen No. 4 (62.87 mg/plant, dry weight 76.04 g) (Figure 3c). The biomass of the underground part was the highest in specimen No. 1 (dry weight 126.81 g, rosavins 659.40 mg/plant, cinnamyl alcohol 251.07 mg/plant), and the lowest in specimen No. 8 (dry weight 52.92 g, rosavins 749.16 mg/plant, cinnamyl alcohol 115.01 mg/plant). Variation of the rosavins:cinnamyl alcohol ratio was greater in the root (from 0.99 to 28.44, coefficient of variance 150.33%) than in the rhizome (from 1.48 to 12.77, coefficient of variance 61.33%). The highest ratio of rosavins to cinnamyl alcohol was noted in the rhizome of specimen No. 4 (28.44), at 10.68 in the root. The lowest ratio was found in the rhizome of specimen No. 5 (0.99), at 3.28 in the root (Figure 3d).

Rosavins were always found to be the predominant phenylpropanoids in all specimens, in both the root and rhizome, except for specimen No. 5, whose rhizome had a concentration of cinnamyl alcohol 50% higher than that of phenylpropanoids (Figure 3e,f). The highest relative abundance of rosavins among all phenylpropanoids was detected in the roots and rhizomes of specimen No. 4 (about 90%), and the lowest in the roots of specimen No. 3 (58%) and the rhizome of specimen No. 5 (48%). A comparison of the rosarin:rosavin:rosin ratio among the specimens revealed that among rosavins, rosavin was predominant, except for specimens No. 1 (root) and No. 3 (root and rhizome), where rosin predominated. The rosarin:rosavin:rosin ratio was 1:1.2–3.2:1.3–2.3 in the root and 1:1.1–3.9:0.7–1.9 in the rhizome.

### 2.4. The Phenylethanoid Content

**Figure 4:**
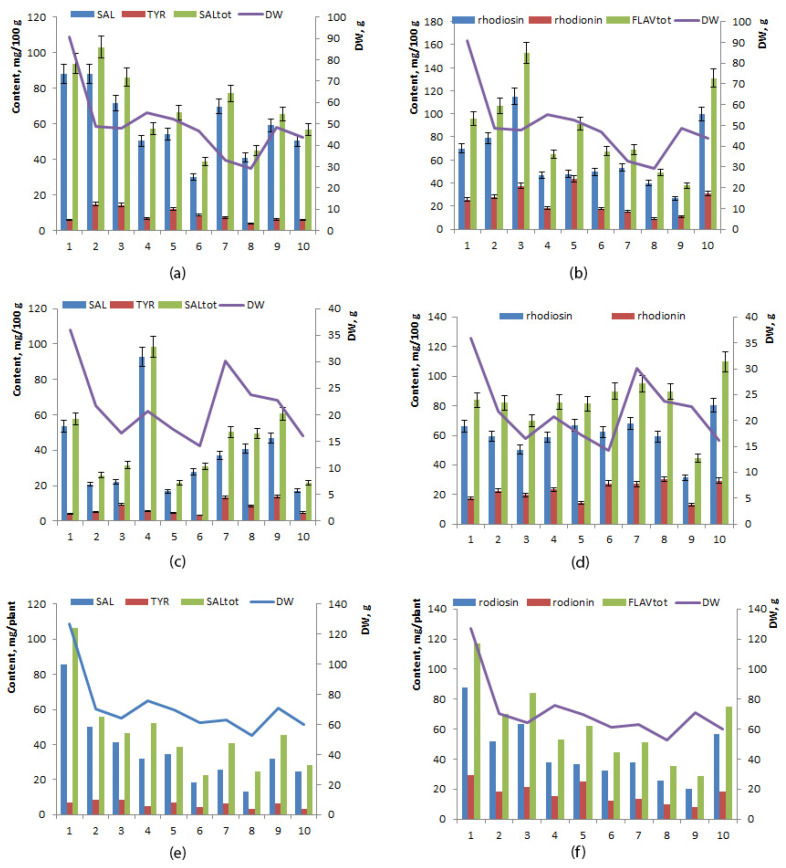
Concentrations of phenylethanoids (**a**,**c**,**e**), flavonoids (**b**,**d**,**f**), and growth parameters of 6-year-old *R. rosea* plants cultivated in the forest–steppe zone of Western Siberia: (**a**,**b**) in rhizomes, (**c**,**d**) in roots, (**e**,**f**) in the whole plant. The horizontal axis: specimens No. 1–10.

The mean level of phenylethanoids was significantly higher in the rhizome than in the root (60.33 ± 6.01 and 37.56 ± 7.35 mg/100 mg, respectively), mainly owing to the presence of more salidroside (Table 2). Interindividual variation of concentrations of salidroside and tyrosol was high in both the root and rhizome: in the root, salidroside ranged between 16.76 and 92.65 mg/100 g (coefficient of variance 61.85%), and tyrosol ranged between 4.23 and 13.78 mg/100 g (coefficient of variance 52.43%), whereas in the rhizome, salidroside ranged from 30.30 to 88.04 mg/100 g (coefficient of variance 31.51%) and tyrosol ranged between 5.96 and 14.96 mg/100 g (coefficient of variance 44.29%). No direct or inverse correlation was found between the yield of roots, of rhizomes, or of the whole plant and levels of salidroside, tyrosol, or phenylethanoids (*p* ≤ 0.05). The highest concentration of salidroside was noted in specimen No. 4 in the root (92.65 mg/100 g), and the lowest in the rhizome of specimens No. 5 (16.76 mg/100 g) and No. 10 (16.96 mg/100 g) (Figure 4a,c). The highest yield of phenylethanoids per plant was registered in specimen No. 1 (106.12 mg/plant), and the lowest in specimen No. 6 (22.68 mg/100 g) (Figure 4e). The phenylpropanoids:phenylethanoids ratio varied from 15.60 to 49.14 in the root and from 6.86 to 29.61 in the rhizome.

### 2.5. Flavonoid Content

The mean levels of rhodiosin, rhodionin, and total flavonoids did not differ significantly between the rhizome and root (*p* ≤ 0.05) (Table 2). Interindividual variations in rhodiosin and rhodionin concentrations were higher in the root (range 26.88–115.08 mg/100 g, coefficient of variance 44.43% and 9.14–43.42 mg/100 g, coefficient of variance 47.43%, respectively) than in the root (range 31.40–80.46 mg/100 g, coefficient of variance 21.32%, and 13.19–30.40 mg/100 g, coefficient of variance 27.39%, respectively) (Figure 4b,d,f).

### 2.6. Hydroxybenzoic Acids

**Figure 5:**
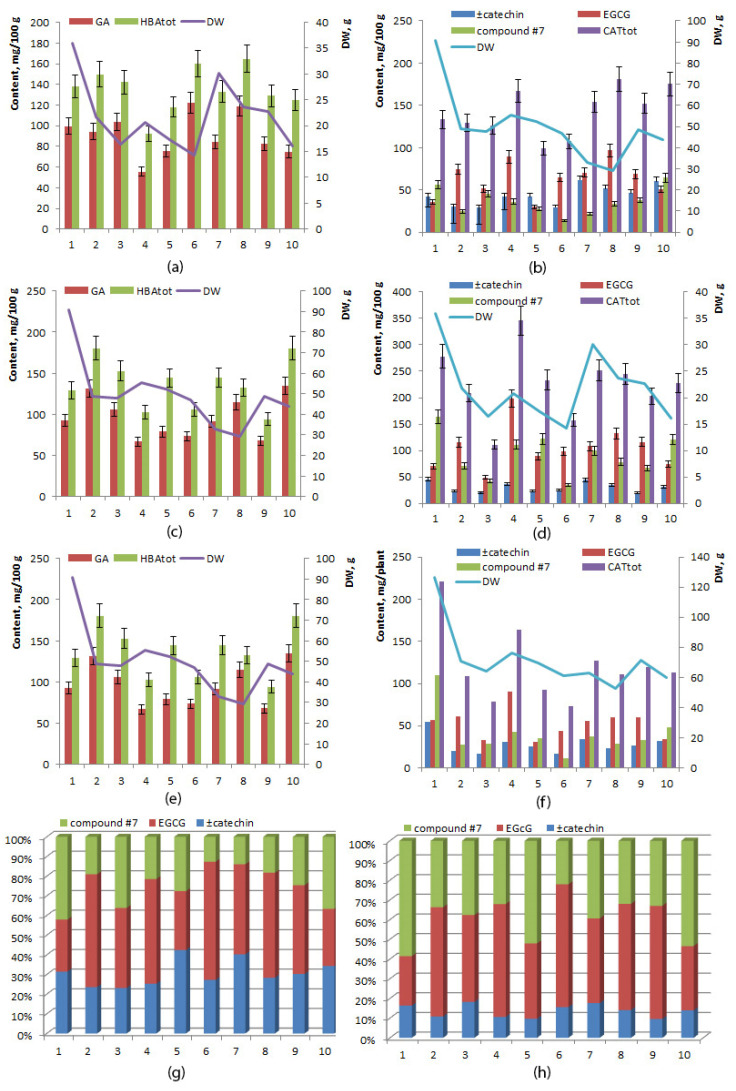
Concentrations of hydroxybenzoic acids (**a**,**c**,**e**) and catechins (**b**,**d**,**f**) in rhizomes (**a**,**b**), in roots (**c**,**d**), and in the whole plant (**e**,**f**); a relative catechin profile (%) of the rhizome (**g**) and root (**h**) of 6-year-old *R. rosea* plants cultivated in the forest–steppe zone of Western Siberia. The horizontal axis: plants (specimens) No. 1–10.

Mean levels of gallic acid did not differ significantly between the rhizome and root (*p* ≥ 0.05), and the same was true for total hydroxybenzoic acids (Table 2). Interindividual variation of total concentration of hydroxybenzoic acids was higher in the rhizome (range 94.15–180.48 mg/100 g, coefficient of variance 22.08%) than in the root (range 92.04–164.57 mg/100 g, coefficient of variance 15.70%) (Figure 5a,c,e).

### 2.7. The Catechin Content

The mean total concentration of catechins was significantly higher in the root than in the rhizome (226.12 ± 20.27 and 142.59 ± 8.83 mg/100 g, respectively), mainly owing to a larger amount of epigallocatechin gallate and of unidentified compound No. 7 (Table 2). Interindividual variation of total concentration of catechins was higher in roots (range 110.87–345.87 mg/100 g, coefficient of variance 28.35%) than in the rhizome (range 99.12–180.68 mg/100 g, coefficient of variance 19.62%) (Figure 5b,d). The highest total concentration of catechins was found in the root of specimen No. 4 (345.87 mg/100 g), and the lowest in the rhizome of specimen No. 5 (99.12 mg/100 g) (Figure 5f). The relative quantity of unidentified compound No. 7 among all catechins was 61% in the root of specimen No. 1, 53% in the root of specimen No. 5, and 55% in the root of specimen No. 10 (Figure 5g,h).

## 3. Discussion

### 3.1. Growth Parameters

We showed that under the conditions of the temperate climate in the forest–steppe zone of Western Siberia, *R. rosea* plants obtained via propagation in vitro are characterized by active growth throughout the entire study period (six years). The highest biomass of roots and rhizomes (dry weight) was noted in the 6th year of cultivation and amounted to 71.56 ± 12.72 g. The rhizome:root ratio (dry weight) changed over the years. The highest rhizome:root ratios (dry weight) were observed in the fourth and sixth years of cultivation (1:0.58 and 1:0.44, respectively); in the same years, the largest annual increase in biomass was documented (362% and 315%, respectively). According to our results, the largest annual increase in the underground part is explained by the growth of the rhizome of *R. rosea*. Under the conditions of introduction into the Republic of Mari El (Russia), *R. rosea* is capable of producing an underground mass (dry weight) of 0.65 to 6.72 tons per hectare (in terms of one plant: 13 to 134 g) [35]. That study indicates that the increase in the biomass of the *R. rosea* underground part depends on the geographical origin, on the agricultural background, on the duration of plant cultivation, on the size of the planted cuttings, and on sexual differentiation. The biomass of the underground part (dry weight) of *R. rosea* cultivated at 1580 m a.s.l. in eastern Austria after 6 years is 194.5 ± 16.2 g, with a rhizome:root ratio of 1:0.75 ± 1:0.06 [23].

According to Peschel et al. [23], after nine years of growing of *R. rosea* in high mountains, the biomass of the rhizome exceeds that of the root. Previously, this pattern has been documented only for plants grown under lowland conditions [21,22,36]. In addition, those authors have stated that the particulation of the rhizome in *R. rosea* cultivated under lowland conditions occurs earlier (by ~5 years) than when grown under more extreme highland conditions. Peschel et al. [23] report that, both in terms of composition and yield, a producer of *R. rosea* raw materials should be interested in plant varieties and conditions that ensure a high proportion of the rhizome. Some authors also report decay of underground parts starting from the fifth year of life, a change in the rhizome/root ratio, a decrease in the yield, and drying-related expenses (rotten parts are often saturated with moisture) for the cultivated plants. Such “ageing” of *R. rosea* appears to begin much earlier in cultivated plants than in wild plants, which grow under more severe conditions and are characterized by a long development cycle [36,37]. Previously, we have found that rhizomes of *R. rosea* in the fourth year of cultivation are easily divided into separate segments (particles) and can be used as mother plants for establishing plantations [25]. Zaprometov [38] says that the localization of glycosides in the underground part of *R. rosea* may be related to their participation in the formation of lignin and suberin. Cinnamyl alcohol and its oxidation products are involved in the formation of three-dimensional structure of lignin. This is important for vegetative propagation of *R. rosea* via rhizome particulation, which is often observed under natural conditions.

### 3.2. The Phenylpropanoid Content

We showed that in six-year-old *R. rosea* plants cultivated in the temperate climate of Western Siberia, the level of phenylpropanoids was on average 1.28 times higher in the roots than in the rhizome, in contradiction to most studies. According to the literature data, the concentration of phenylpropanoids is 1.5–4 times higher in the rhizome, in both wild and cultivated plants [21]. According to our data, the levels of rosavins and of cinnamyl alcohol and the rosavins:cinnamyl alcohol ratio varied significantly among the studied specimens. For instance, the rosavins:cinnamyl alcohol ratio in roots varied from 0.99 to 28.44. It has been reported that, regardless of season and age, specimens of *R. rosea* from the Alps and the Pyrenees have higher mean rosavins:cinnamyl alcohol ratios, between 9 and 15, as compared to plants from northern Europe (between 3 and 8) [23]. It has been previously reported that among rosavins, the greatest changes in the rhizome and roots of introduced *R. rosea* plants (from Gorny Altai) are observed in rosavin, and cinnamyl alcohol is the predominant phenylpropanoid in all tested specimens (up to 58%, in terms of rosavin); a detailed analysis of rosavins revealed that in all specimens, except for the roots from the second year of cultivation, the predominant phenylpropanoid is rosavin [25]. According to other researchers, the concentration of cinnamyl alcohol is 5–30% of that of rosavins (cinnamyl alcohol and rosavins were quantified as cinnamyl alcohol and rosavin, respectively) [22]. According to our findings, in the analyzed specimens, the predominant phenylpropanoid is also rosavin, except for specimens No. 1 (root) and No. 3 (root and rhizome), in which the main phenylpropanoid was rosin. Our present assays indicate that some *R. rosea* plants feature high rhizome biomass with a moderate concentration of phenylpropanoids (specimen No. 1) or vice versa (specimen No. 4). Despite the wide range of values, all 10 tested specimens (underground part) met the minimum requirements of the United States Pharmacopeia [6] for rosavins (0.3%): 403.92–1415.65 mg/100 g, and of the Russia State Pharmacopoeia for the average value of rosavins in roots: (1%) [7], i.e., 1081.43 mg/100 g.

Concentrations of secondary metabolites in plant raw materials can be influenced by various factors: the plant genotype, soil and climatic conditions, and agricultural practices as well as methods of extraction and quantitative analysis. For instance, Peschel et al. [23] have found that the harvest season has a greater influence on the level of rosavins during growth in temperate climates than do the origin and duration of cultivation. Harvesting is usually completed at the end of the growing season, but this choice may not be optimal for *R. rosea*. Instead, Peschel et al. [23] recommend harvesting in the spring before or at the time of germination. This idea is supported by data on a rather high concentration of rosavin in vegetative buds (comparable to that in the rhizome according to ref. [20]). Despite the lower level of rosavins, there are some practical benefits to “late-season” harvesting: greater biomass growth and lower costs of harvesting, cleaning, and drying. Kołodziej and Sugier [20] report that roseroot harvested after only three years of growth contains significantly lower amounts of phenylpropanoids and phenylethanoids in underground parts of the plants than when harvested after the fourth, fifth, or sixth year. Because these phenolics and glycosides are the major active ingredients of *R. rosea*, this switching to an earlier harvest (before the fourth year, or in appropriate cases, in the third year) may have an effect on the quality of the harvested raw material.

Alperth et al. [30] conducted a comparative analysis of various extraction methods applied to the rhizome of roseroot: conventional ethanol extraction (35%, 70%, and 96%, *v*/*v*) and accelerated solvent (85% methanol) extraction. It was revealed that methanolic accelerated solvent extraction is more efficient than conventional ethanol extraction and produces the highest yield of all studied substances (including rosavins, salidroside, and flavonoids), except for cinnamyl alcohol. For example, the highest concentration of rosavin in a specimen from Austria (High Tauern region) after the accelerated solvent extraction was 1565.06 mg/100 g, whereas after ethanol extraction, 204.74 mg/100 g. Kučinskaitė et al. [29] have demonstrated that switching from 40% (*v*/*v*) ethanol as the extraction solvent to 70% ethanol leads to a significant increase in the amount of rosavin extractable from the analyzed material (introduced and wild plants), whereas changes in the concentrations of rosarin and rosin were negligible. It has been found that the freeze-drying method increases the concentration of all phenylethanoids and phenylpropanoids in rhizomes as compared with conventional drying at 70 °C [31].

### 3.3. The Phenylethanoid Content

The literature describes a ≥2-fold excess of rosavins relative to salidroside, regardless of a plant part used, sampling site, or extraction methods used. The only exception is the specimen (described by Malnoe et al. [39]) from the Swiss Alps, in which the level of salidroside exceeds that of rosavins. In our work, the phenylpropanoid:phenylethanoid ratio varied from 15.60 to 49.14 in the root and from 6.86 to 29.61 in the rhizome. Overall, the levels of salidroside and tyrosol were not high in our analyzed specimens and amounted to 16.76–92.65 and 4.23–14.96 mg/100 g, respectively. According to the literature, concentrations of salidroside and tyrosol in specimens collected in different regions of China vary between 1.3–11.1 and 0.3–2.2 mg/g, respectively [40]. The highest salidroside content was registered in 16-year-old *R. rosea* plants from Norway: 51.0 mg/g [31]. Those authors also stated that long-term cultivation promotes the accumulation of biologically active compounds (rosavins and salidroside) in *R. rosea*. It is reported that the dynamics of the level of phenylethanoids are linked to the stages of development of above-ground biomass and the sex of the plant. In general, the concentration of phenylethanoids is higher in male specimens than in female ones [41]. It has been demonstrated that after introduction, the level of phenylethanoids diminishes in the third year owing to intensive plant growth [42].

### 3.4. The Flavonoid Content

The antioxidant properties of *R. rosea* are determined by flavonoids, of which rhodionin and rhodiosin are the two major flavonoids. Recently, due to suppression of postprandial elevation of the blood triglyceride level and owing to their hepatoprotective and prolyl endopeptidase- and neuraminidase-inhibitory effects [43,44,45], the two compounds showed promise as pharmaceuticals or nutritional supplements [46]. Flavonoids may not only contribute to some activities but also may represent an additional analyte for ensuring identity, purity, and consistent composition of medicinal products [5,47]. Regarding *R. rosea*, the isolated flavonoids are usually glycosides of kaempferol, gossypetin, and herbacetin. More than 20 flavonoids in this species have been described, including tricin, herbacetin, gossypetin, and their glycosides found in leaves/flowers/aerial parts as well as flavonolignans and herbacetin found in underground parts, i.e., in the rhizome or root [48,49,50].

Concentrations of individual flavonoids vary depending on origin, part of the plant analyzed, and the solvent used for extraction. For example, the level of rhodiosin in various specimens of *R. rosea* from Austria is 8.72 to 25.90 after ethanol extraction and 320.38 to 619.65 mg/100 g after accelerated solvent extraction, whereas the concentration of rhodionin proved to be 1.55–5.61 and 81.92–175.63 mg/100 g, respectively, with similar extraction methods [30]. In general, rhodiosin tends to be a major phenolic compound along with herbacetin, rhodionin, and kaempferol [51,52,53]. According to our data, the main flavonoid was also rhodiosin. Peter Zomborszki et al. [5] have shown that, on average, rhizome and root extracts of a nine-year-old *R. rosea* plant (grown in the eastern Alps) contain 1800 and 3100 µg/mL of flavonoids, respectively. At the same time, root extracts contained more of total flavonoids than rosavins, and the ratio of rosavins to total flavonoids differed significantly between the rhizome and roots (1.4 versus 0.4, respectively). Those authors stated that the total amount of flavonoids usually exceeds that of salidroside, which is a common standardization parameter for all *Rhodiola* species. According to our findings, the total concentration of flavonoids did not exceed 152.54 mg/100 g and did not differ significantly between the roots and rhizome. The level of rosavins significantly exceeded the total level of flavonoids, while the concentration of phenylethanoids was ≥2 times lower. Ratios of rosavins/total flavonoids and phenylethanoids/total flavonoids were on average 8.83 and 0.89 in the rhizome and 13.05 and 0.59 in the root, respectively.

### 3.5. Concentrations of Hydroxybenzoic Acids and Catechins

According to Olennikov et al. [54], the main class of phenolic compounds in the underground organs of *R. rosea* is catechins: their concentrations are 10.84 and 61.30 mg/g in the roots and rhizome, respectively. Other investigators have shown that the level of catechins in the root can vary from 4.6 mg/g (in specimens from Poland [55]) to 20 mg/g (in specimens of Indian origin [56]). Judging by our data, the total concentration of catechins is on average 1.61 times higher in the root of *R. rosea* than in the rhizome (mainly because of compound No. 7) and amounts to 226.12 ± 20.27 mg/100 g.

Among all phytochemicals, phenolics have aroused considerable interest because of their various biological activities, such as antioxidant, anti-inflammatory, antiviral, and antimicrobial properties [57,58,59]. Phenolic acids play a leading role in the lignification process [60]. Nonetheless, according to our data, the total concentration of hydroxybenzoic acids does not differ significantly between the rhizome and root. Zhang et al. [61] have shown that *R. rosea* extract of free phenolics is rich in phenolics and flavonoids. Among all the detected phenolic compounds, the concentration of epigallocatechin gallate was the highest, followed by gallic acid, epigallocatechin, and catechin. Those authors revealed that *R. rosea* free phenolics have good potential for the development of auxiliary antioxidant and therapeutic agents for cancer. According to our findings, in the rhizome, the dominant phenolic compound was gallic acid (95.72 mg/100 g), whereas in roots, major phenolic compounds were epigallocatechin gallate (105.10 mg/100 g), gallic acid (91.10 mg/100 g), and compound No. 7 (90.81 mg/100 g).

## 4. Materials and Methods

### 4.1. Plant Material and Propagation

*R. rosea* seeds were collected from natural habitats: the Altai Republic (Russia), the southern slopes of the Iolgo ridge, and the Karakol Lakes, at an altitude of 1800–2000 m a.s.l. Thirty *R. rosea* seeds were used for in vitro introduction and micropropagation. The in vitro propagation of *R. rosea* was carried out at the Biotechnology Laboratory of the Central Siberian Botanical Garden, SB RAS (CSBG) according to a previously developed methodology [62,63]. The cultivation of plants in open ground was performed according to published procedures [25,26]. To conduct an experiment to assess the parameters of growth and accumulation of biologically active compounds, 100 plants propagated in vitro were randomly selected, acclimatized to ex vitro conditions, and planted on an experimental site (introduction population). Ten plants from the introduction population were collected and analyzed annually from 2018 to 2022.

### 4.2. Growth Conditions

The introduction site is located on the territory of the CSBG, on the right bank of the Novosibirsk Reservoir, 25 km from downtown Novosibirsk (Russia). Geomorphologically, the CSBG territory occupies the second and third terraces above the Ob River floodplain, which is composed of ancient alluvial sandy and sandy loam deposits. The average altitude of the terraces is 150–200 m a.s.l. The soils are light gray, forest-type, and medium thick. The humus content is 3–5%; the average concentration of mobile nitrogen is 20–40 mg/kg; the phosphorus level is 10–15 mg/100 g; the soil pH is 5.6–6.0.

### 4.3. Harvest and Drying

The plants were harvested at the end of the growing season (5th year of cultivation—30 September 2021, 6–10 October 2022). Rhizomes and roots were washed, then the roots were separated from rhizomes and cut into pieces (maximum thickness of 1 cm) (Figure 1). The roots and rhizomes were not cleansed of periderm. Dry weights of roots and rhizomes were recorded for each plant after drying to constant weight via warm-air ventilation at 45 °C. Dry samples were stored in paper bags under dark dry conditions at 20–22 °C.

### 4.4. Extraction and HPLC Analysis of Phenolic Compounds

Double extraction was performed to extract phenolic compounds. An exactly weighed sample (0.2 g) of the crushed material was extracted via maceration with 10 mL of aqueous 50% ethanol for 5 days, and then with 20 mL of 70% ethanol for 60 min in a water bath at 60–70 °C. The combined extract was concentrated (via evaporation) to 20 mL and passed through a membrane filter with a pore diameter of 0.45 μm. HPLC analysis of the aqueous–ethanol extracts was carried out using an Agilent 1200 system with a diode array detector and the software program ChemStation for data processing (Agilent Technologies, Santa Clara, CA, USA). Chromatographic separation was performed at 25 °C on a Zorbax SB-C18 column (4.6 × 150 mm, 5 μm internal diameter) (Agilent Technologies, Santa Clara, CA, U SA). The mobile phase consisted of MeOH (solvent A) and 0.1% orthophosphoric acid in water (solvent B). The gradient was started with an A–B mixture at 22:78 (*v*/*v*) followed by a linear gradient to 70:30 (*v*/*v*) for the first 30 min, and then to 100:0 (*v*/*v*) from minute 30 to minute 32. A return of the mobile phase to 22:78 (*v*/*v*) was implemented from minute 32 to minute 36. The flow rate was set to 1 mL/min. The sample injection volume was 10 μL. Tracking of chromatograms was conducted by means of absorbance at 220, 255, 270, 290, 325, 340, 350, 360, and 370 nm. Phenolic compounds were quantified via the external standard method. Quantification of hydroxybenzoic acids, catechins, flavonoids, tyrosol, salidroside, and rosavins was carried out according to the calibration curve for gallic acid (GA), (+)-catechin (Sigma-Aldrich, St. Louis, MO, USA), epigallocatechin gallate (Teavigo, Gevelsberg, Germany), astragalin (Sigma-Aldrich, St. Louis, MO, USA), tyrosol (Pharmaffiliates Analytics & Synthetics (P) Ltd., Panchkula, India), salidroside, and rosavin (Aobious, Gloucester, MA, USA), respectively, in the concentration range of 10–300 µg/mL.

The limit of detection (LOD) with a signal-to-noise ratio of 3.3 or higher (LOD = 3.3 ∗ σ/S, µg/mL) and the limit of quantification (LOQ) with a signal to noise ratio of 10 or higher (LOQ = 10 ∗ σ/S, µg/mL) were determined for salidroside and tyrosol. The linearity was determined using 5 different concentrations per reference standard in the range of 10 µg/mL to 300 µg/mL with a linear relationship. Each sample was measured in duplicate. For salidroside, the coefficient of determination (R-squared) or correlation coefficient, R^2^ = 0.9971, regression equation y = 7.935x + 55.143, LOD = 16.2 µg/mL. LOQ = 49.2 µg/mL. For tyrosol, R^2^ = 0.998, regression equation y = 5.9321x + 33.843, LOD = 13.1 µg/mL. LOQ = 39.6 µg/mL.

Concentrations of phenolic compounds were expressed in mg per 100 g of air-dried weight. Each sample was analyzed as three technical replicates.

### 4.5. Statistical Analysis

All the data were processed in the software program STATISTICA 6.0 (Statsoft Inc., Tulsa, OK, USA), are reported as mean ± standard error (SE) of three replicates, and were compared using ANOVA followed by Duncan’s multiple-range test. Differences between the means were considered statistically significant at *p* ≤ 0.05.

In this work, we also summarized the total level of rosavins (rosarin + rosavin + rosin), the total concentration of phenylpropanoids (rosavins + cinnamyl alcohol), the total level of phenylethanoids (salidroside + tyrosol), the total concentration of flavonoids (rhodiosin + rhodionin), the total level of hydroxybenzoic acids (gallic acid + hydroxybenzoic acid derivative), and total concentration of catechins [(±)-catechin + epigallocatechin gallate + compound No. 7].

Ratios were calculated for some comparisons: rosarin–rosavin–rosin–cinnamyl alcohol (with rosarin set to 1.0), rosavins–cinnamyl alcohol (with cinnamyl alcohol set to 1.0), rosavins–salidroside (with salidroside set to 1.0), rosavins–flavonoids, and salidroside–flavonoids (with flavonoids set to 1.0).

## 5. Conclusions

In this paper, we analyzed interindividual variation between growth parameters and between levels of secondary metabolites as well as the productivity of *R. rosea* plants (originating in the Altai Mountains) cultivated under the conditions of the forest–steppe zone of Western Siberia. In the sixth year of cultivation of *R. rosea* in the temperate climate, the level of phenylpropanoids was on average 1.28 times higher in the roots than in the rhizome and amounted to 1273.72 mg/100 g. Despite the wide range of values, all 10 specimens (underground part) met the minimum requirements of the United States Pharmacopeia [6] for rosavins (0.3%) and of the Russia State Pharmacopoeia for the average level of rosavins in the roots: (1%) [7]. Total concentration of phenylethanoids in underground organs was not high and ranged from 21.36 to 103.00 mg/100 g. Overall, high interindividual variation was demonstrated both in growth characteristics and in the concentrations of secondary metabolites in *R. rosea* under our cultivation conditions. It was noted that the contents of phenylpropanoids, phenylethanoids, and catechins significantly depends on the plant part being analyzed (*p* ≤ 0.05).

## Figures and Tables

**Table 1:** The yield and rhizome:root ratio (dry weight; DW) of 2–6-year-old specimens of *R. rosea* cultivated in the forest–steppe zone of Western Siberia (Russia), n = 10.

Year of Cultivation	Total DW, g		DW Increase over the Last Year, %	Rhizome: Root Ratio, DW
	Mean ± SE	CV, %		
Year 2	2.93 ± 0.35	27.72	–	1:0.71 ^1^
Year 3	4.21 ± 0.46	21.63	144	1:0.75 ^1^
Year 4	15.22 ± 1.37	19.54	362	1:0.58 ^1^
Year 5	22.74 ± 2.86	24.68	149	1:0.78
Year 6	71.56 ± 6.49	17.78	315	1:0.44

^1^ data published earlier by Erst et al. [25].

**Table 2:** Characteristics and levels of the phenolic compounds (mg/100 g) detected via HPLC in an aqueous–ethanol extracts from rhizomes and roots of *R. rosea* (presented as $\frac{rhizome}{root}$ in each row).

ID	Compound	SCh: λmax, nm	t_R_, min	Specimen ID										Mean ± SE	CV, %
				1	2	3	4	5	6	7	8	9	10		
hydroxybenzoic acids															
1	GA	216, 272	2.3	92.74 99.38	131.55 94.42	105.96 103.55	66.71 55.41	78.87 75.51	73.21 122.24	91.12 84.14	114.51 118.98	67.83 82.50	134.67 74.90	95.72 ± 7.96 a 91.10 ± 6.57 a	26.29 22.80
2	HBA derivative	220, 270	2.6	36.29 38.37	48.71 55.42	46.70 38.50	36.06 36.63	65.22 42.61	32.54 37.78	53.74 48.87	17.59 45.59	26.32 46.31	45.80 50.08	40.90 ± 4.40 a 44.02 ± 1.99 a	34.05 14.28
	HBA_tot_			129.03 137.75	180.26 149.84	152.66 142.05	102.77 92.04	144.08 118.12	105.74 160.02	144.86 133.01	132.10 164.57	94.15 128.81	180.48 124.98	136.61 ± 9.54 a 135.12 ± 6.71 a	22.08 15.70
phenylethanoids															
3	SAL	222, 276	4.8	88.00 53.46	88.04 20.78	71.68 22.27	50.55 92.65	54.15 16.76	30.30 27.69	69.75 37.20	41.09 40.83	59.18 46.98	50.58 16.96	60.33 ± 6.01 a 37.56 ± 7.35 b	31.51 61.85
4	TYR	220, 275	6.3	5.96 4.23	14.96 4.93	14.46 9.37	6.90 5.57	12.14 4.60	8.66 3.27 ^1^	7.37 13.08	3.83 ^1^ 8.62	6.35 13.77	6.09 4.80	8.67 ± 1.21 a 7.23 ± 1.20 a	44.29 52.43
	SAL_tot_			93.96 57.69	103.00 25.71	86.14 31.64	57.45 98.22	66.29 21.36	38.96 30.96	77.12 50.28	44.92 49.45	65.53 60.75	56.67 21.76	69.00 ± 6.60 a 44.78 ± 7.55 b	30.27 53.34
catechins															
5	±catechin	204, 230 sh., 280	5.5	41.93 45.48	30.49 22.66	29.01 20.17	42.30 36.72	42.03 22.56	29.28 24.54	61.80 44.37	51.24 34.60	46.04 19.47	60.14 31.55	43.43 ± 3.75 a 30.21 ± 3.09 a	27.30 32.38
6	EGCG	204, 230 sh., 275	7.5	35.30 69.87	74.07 115.82	51.23 48.92	89.02 198.26	29.71 89.26	64.53 98.15	70.46 108.17	96.48 132.11	68.77 116.36	50.84 74.12	63.04 ± 6.81 b 105.10 ± 13.04 a	34.16 39.25
7	No. 7	228, 277	8.2	56.14 163.09	24.58 70.50	45.48 41.74	35.94 110.89	27.38 121.61	13.56 34.76	21.50 99.15	32.96 78.29	37.51 66.83	64.20 121.19	35.93 ± 4.97 b 90.81 ± 12.59 a	43.73 43.84
	CAT_tot_			133.37 278.44	129.14 208.98	125.72 110.83	167.26 345.87	99.12 233.43	107.37 157.45	153.75 251.69	180.68 245.00	152.32 202.66	175.18 226.85	142.39 ± 8.83 b 226.12 ± 20.27 a	19.62 28.35
phenylpropanoids															
8	rosarin	253.00	13.9	101.51 134.34	193.41 169.07	110.48 65.68	243.74 264.45	126.46 168.92	180.72 196.21	134.02 143.97	260.54 357.26	178.81 266.76	271.97 332.13	180.17 ± 19.77 a 209.88 ± 29.30 a	34.69 44.14
9	rosavin	253.00	14.9	167.76 308.24	340.78 506.04	147.51 78.25	951.08 850.65	134.11 336.22	393.47 426.33	196.02 345.73	510.76 904.44	316.28 763.17	339.88 606.00	349.76 ± 77.09 a 512.51 ± 84.08 a	69.70 51.88
10	rosin	253.00	15.2	197.07 213.16	281.20 370.43	184.04 149.73	425.26 500.54	97.44 281.13	223.54 283.09	148.50 182.50	298.09 580.21	174.57 470.07	306.97 559.63	233.67 ± 30.12 a 359.05 ± 50.52 a	40.77 44.50
11	CA	253.00	19.0	179.54 244.67	326.78 217.93	225.55 198.94	56.96 151.32	363.44 239.64	154.70 115.21	180.09 127.33	260.63 164.01	194.94 117.45	369.01 346.37	231.16 ± 31.50 a 192.29 ± 23.01 a	43.10 37.85
	ROS_tot_			466.34 655.75	815.39 1045.53	442.02 293.65	1620.09 1615.63	358.01 786.27	797.72 905.62	478.54 672.20	1069.39 1841.91	669.66 1499.99	918.83 1497.76	763.60 ± 120.25 b 1081.43 ± 159.89 a	49.80 46.75
	PP_tot_			645.89 900.41	1142.17 1263.47	667.57 492.60	1677.05 1766.96	721.45 1025.91	952.42 1020.83	658.63 799.54	1330.01 2005.92	864.60 1617.45	1287.83 1844.13	994.76 ± 11.59 b 1273.72 ± 160.65 a	35.47 39.88
flavonoids															
12	rhodiosin	277, 333, 385	21.5	70.09 66.37	79.09 59.53	115.08 50.32	46.39 58.95	47.88 67.10	49.63 62.38	53.28 67.89	39.77 59.18	26.88 31.40	99.94 80.46	62.80 ± 8.82 a 60.36 ± 4.07 a	44.43 21.32
13	rhodionin	277, 333, 386	22.1	25.61 17.58	27.88 22.56	37.47 19.67	18.56 23.43	43.42 14.31	17.78 27.55	15.48 27.07	9.14 30.39	10.84 13.20	30.78 29.33	23.70 ± 3.58 a 22.51 ± 1.95 a	47.83 27.39
	FLAV_tot_			95.71 83.95	106.97 82.09	152.54 69.99	64.94 82.38	91.31 81.40	67.42 89.93	68.77 94.96	48.91 89.57	37.71 44.60	130.72 109.80	86.50 ± 11.44 a 82.87 ± 5.38 a	41.83 20.51
	Total			1097.95 1458.25	1661.55 1730.09	1184.63 847.10	2069.47 2385.47	1122.26 1480.22	1271.90 1459.20	1103.13 1329.48	1736.63 2554.51	1214.31 2054.26	1830.88 2327.52	1429.27 ± 113.52 a 1762.61 ± 173.59 a	25.13 31.13

SCh: Spectral characteristics, t_R_: retention time, PP_tot_: sum of phenylpropanoids; ROS_tot_: sum of rosavins; CA: cinnamyl alcohol; SAL_tot_: sum total of phenylethanoids; SAL: salidroside; TYR: tyrosol; FLAV_tot_: sum of flavonoids; CAT_tot_: sum of catechins; EGCG: epigallocatechin gallate; HBA_tot_: sum of hydroxybenzoic acids; GA: gallic acid; No. 7: unidentified compound No. 7. Values followed by the same letter within each row are not significantly different for the contents of phenolic compounds in rhizome and root (*p* ≥ 0.05) according to Duncan’s multiple-range test. ^1^—values below the limit of quantification (LOQ).

## Data Availability

All data necessary for reproducing our results are included in this published article. Raw data are available upon request.
